# Supplementary material for: Risk factors for vitamin D deficiency in Abu Dhabi Emirati population
Source: PLoS One. 2022 Feb 15;17(2):e0264064. doi: 10.1371/journal.pone.0264064 (PMC8846532; doi:10.1371/journal.pone.0264064)
Supplement: S1 Questionnaire — (DOC) [file pone.0264064.s002.doc]

استبيان

- **الرقم التسلسلي للمشارك في البحث**
- **العمر_______**

| انثى |  |
| --- | --- |
| ذكر |  |

- **الجنس**
- **مستوى التعليم**

| أمي |  |
| --- | --- |
| ابتدائي |  |
| اعدادي |  |
| ثانوي |  |
| جامعي |  |
| ماجستير , دكتوراه |  |
| لا أعرف |  |

- **النظام الغذائي:**
  - **كم عدد الحصص الغذائية التي تأكلها يوميا من منتجات الألبان (الحليب(كأس واحد)- زبادي((كأس واحد)) - اللبن(كأس واحد) - الجبن(شريحتان= 40 جرام) او غيرها ) ؟**

| 0 وجبه /حصة |  |
| --- | --- |
| 1 وجبه /حصة |  |
| 2 وجبه /حصة |  |
| 3 وجبه /حصة |  |
| >3 وجبه /حصة |  |

- - كم عدد الحصص الغذائية الغنية بفيتامين دال (زيت كبد سمك الحوت(ملعقة واحدة)- التونة (نصف كوب) -السلمون(نصف كوب) –البيض(عدد 2)) التي تأكلها يوميا ؟

| 0 وجبه /حصة |  |
| --- | --- |
| 1 وجبه /حصة |  |
| 2 وجبه /حصة |  |
| 3 وجبه /حصة |  |
| >3 وجبه /حصة |  |

- - هل سبق تشخيصك بالإسهال المزمن ؟ نعم لا
- **الفيتامينات**
  - هل كنت تتناول أي نوع من الفيتامينات قبل عمل فحص وقاية الطبي؟ نعم لا

اذا كانت الاجابة بنعم كم حبه من الفيتامينات تأخذ فاليوم ؟_______

- - هل كنت تتناول فيتامين دال قبل عمل فحص وقاية الطبي؟ نعم لا

اذا كانت الاجابة بنعم ما هو النوع و الجرعة التي تأخذها ؟_______

- - هل كنت تتناول كالسيوم قبل عمل فحص وقاية الطبي؟ نعم لا

اذا كانت الاجابة بنعم كم عدد الحبوب التي تتناولها في اليوم ؟_______

- **الألتزام بأخذ العلاج :**
- عندما تم و صف فيتامين دال لك هل كنت تأخذه بانتظام كما وصف لك ؟ نعم لا
- هل نسيت أخذ اي حبه من حبوب العلاج ؟ نعم لا

اذا كانت الاجابة بنعم كم عدد الحبوب التي نسيت أخذها ؟_______

- هل توقفت عن أخذ حبوب فيتامين قبل نهاية فترة العلاج ؟ نعم لا
- هل تناولت حبوب الكالسيوم مع فيتامين دال خلال فترة العلاج ؟

اذا كانت الاجابة بنعم كم عدد الحبوب التي تتناولها في اليوم ؟_______

- **التعرض لأشعه الشمس:**
  - هل تعرض نفسك للشمس كاشفا ذراعيك وساقيك؟

إذا كانت الإجابة بنعم, كم من الوقت يوميا(بالدقائق) تقضيه معرضا للشمس في الشهر الماضي ؟

| عدد الدقائق | الفترة الزمنية |
| --- | --- |
|  | من الساعة 7 الي 9 صباحا |
|  | من الساعة 9 الي 11 صباحا |
|  | من الساعة 11 صباحا إلي1 ظهرا |
|  | من الساعة 1 ظهرا الي 3 ظهرا  من الساعة 3 ظهرا الي 5 عصرا |
|  | من الساعة 5 عصرا الي 7 مساءا |

- - هل ترتدي قميص بلا اكمام او بنطال القصير ؟

| ابدا |  |
| --- | --- |
| نادرا |  |
| احيانا |  |
| اغلب الاوقات |  |
| دائما |  |

- - هل تستخدم الكريم الواقي من اشعة الشمس ؟

| ابدا |  |
| --- | --- |
| نادرا |  |
| احيانا |  |
| اغلب الاوقات |  |
| دائما |  |

- ممارسة الرياضة:
  - في الاسبوع الماضي كم عدد المرات التي مارست فيها الانشطة الرياضيه (المشي ، ركوب الدارجات ،السباحه او غيرها )

| دقيقه في اليوم |  | يوميا |
| --- | --- | --- |
| دقيقه في الأسبوع |  | اكثر عن مره فالأسبوع |
| دقيقه في الأسبوع |  | مره واحده فالأسبوع |
|  |  | نادرا |
|  |  | ابدا |

- ما نوع النشاط البدني الذي تبذله في فتره العمل ؟

| لا أعمل |  |
| --- | --- |
| اقضي معظم الوقت جالسا في المكتب |  |
| اقضي معظم الوقت في المشي او اكون واقفا |  |
| عملي يتطلب بذل مجهود بدني |  |

- **أخرى :**
- هل اشتكيت من اي من الاعراض التاليه قبل اخذ علاج فيتامين دال ؟
  - آلام فالعظام نعم لا
  - آلام فالظهر نعم لا
  - آلام في العضلات نعم لا
  - ضعف وتعب في العضلات نعم لا
- هل اشتكيت من اي اعراض جانبيه بعد اخذ علاج فيتامين دال ؟ نعم لا

اذا كانت الاجابة بنعم الرجاء ذكر الاعراض ---------------

- هل اصبت بكسر فالعظام سابقا ؟ نعم لا

|  | الظهر |
| --- | --- |
|  | الحوض |
|  | أخرى |
|  | لا أعلم |

- هل تم تشخيصك بمرض هشاشة العظام ؟ نعم لا

اذا كانت الاجابة بنعم ما هو الدواء الذي تأخذه ---------------

- هل تم تشخيص اي فرد من عائلتك بمرض هشاشة العظام ؟ نعم لا
- هل أصيبت والدتك بكسر في الحوض سابقا ؟ ؟ نعم لا
- هل تعاني من اي مرض مزمن ؟ نعم لا

اذا كانت الاجابة بنعم الرجاء ذكر المرض------------
