# Supplementary material for: Risk factors for vitamin D deficiency in Abu Dhabi Emirati population
Source: PLoS One. 2022 Feb 15;17(2):e0264064. doi: 10.1371/journal.pone.0264064 (PMC8846532; doi:10.1371/journal.pone.0264064)
Supplement: S2 Questionnaire — (DOCX) [file pone.0264064.s003.docx]

Research questionnaire

- Serial Number :
- Age:
- Sex:

| Female |  |
| --- | --- |
| Male |  |

- Education level:

| Don’t attend school |  |
| --- | --- |
| Complete primary school |  |
| Complete intermediate school |  |
| Complete secondary school |  |
| Complete college |  |
| Complete master or Ph D |  |
| Do not know |  |

- Social status:

| Low |  |
| --- | --- |
| Medium |  |
| High |  |

- Dietary intake:
  - How many servings of diary products(milk, yogurt, cheese, laban) do you get daily?

| 0 serving |  |
| --- | --- |
| 1 serving |  |
| 2 servings |  |
| 3 servings |  |
| >3 servings |  |

| 0 serving |  |
| --- | --- |
| 1 serving |  |
| 2 servings |  |
| 3 servings |  |
| >3 servings |  |

- - How many servings of vitamin D rich food (cod liver oil, tuna, salmon, eggs) do you get daily?

- - Do you take multivitamins? Yes No
  - If yes, how many multivitamin tablets do you take daily? _________
  - Do you take vitamin D supplements or calcium with vitamin D before weqaya screening? Yes No
  - Do you take vitamin D supplements or calcium with vitamin D? Yes No
  - If so, how many IU per day?_______
  - Have you been diagnosed with chronic diarrhea? Yes No
- Sun Exposure
- What time of the day you spend? From ____to _____how much did you spend outdoors?

| Never |  |
| --- | --- |
| Between the hours of 7 and 9am? |  |
| Between 9 and 11 am? |  |
| Between 11am and 1pm? |  |
| Between 1 and 3pm?  Between 3 and 5pm? |  |
| Between 5 and 7pm? |  |

- Do you wear sleeveless shirt or short pants?

| Never |  |
| --- | --- |
| Rarely |  |
| Sometimes |  |
| Most of the time |  |
| always |  |

- Do you use sun block? Yes No

| Never |  |
| --- | --- |
| Rarely |  |
| Sometimes |  |
| Most of the time |  |
| always |  |

- Physical activity

How often do you practice physical activities (walking, cycling, swimming ,or other )

| daily |  |
| --- | --- |
| More than 1 per week |  |
| weekly |  |
| Rarely |  |
| Never |  |

- Did you complain of any of the following Symptoms before receiving vitamin D treatment :
- Bone pain Yes No
- Back pain Yes No
- Muscle ache Yes No
- Muscle weakness Yes No
- Do you complain of any side effects after taking the vitamin d treatment? Yes No
  - If Yes please indicate the symptoms ---------------
- Did you had any fracture ( broken bone ) before? Yes No
- Did any one of your family has been diagnosed with osteoporosis Yes No
- Do you have any Chronic Medical problem Yes No

If yes please indicated the disease ------------------------------
