# Supplementary material for: Risk factors for vitamin D deficiency in Abu Dhabi Emirati population
Source: PLoS One. 2022 Feb 15;17(2):e0264064. doi: 10.1371/journal.pone.0264064 (PMC8846532; doi:10.1371/journal.pone.0264064)
Supplement: Attachment — Submitted filename: response to reviwer.docx [file pone.0264064.s004.docx]

Journal Requirements:

When submitting your revision, we need you to address these additional requirements.

1. Please ensure that your manuscript meets PLOS ONE's style requirements, including those for file naming. The PLOS ONE style templates can be found at
   <https://journals.plos.org/plosone/s/file?id=wjVg/PLOSOne_formatting_sample_main_body.pdf> and 
   <https://journals.plos.org/plosone/s/file?id=ba62/PLOSOne_formatting_sample_title_authors_affiliations.pdf>

Done

2. In your Methods section, please provide a justification for the sample size used in your study, including any relevant power calculations (if applicable).

We calculated the sample size on the basis of 80% power and a 5% two-tailed significance level. We assumed that we would need to explore modest differences of a continuous variable between two groups of unequal size. We therefore calculated the sample size on the basis of detecting a difference of half a standard deviation between two groups the smallest of which comprises 10 of the total. This yielded a required sample size of 350 (<https://epitools.ausvet.com.au/twomeanstwo>). We then doubled this to take into account factors such as refusals and non-response

Please include additional information regarding the survey or questionnaire used in the study and ensure that you have provided sufficient details that others could replicate the analyses. For instance, if you developed a questionnaire as part of this study and it is not under a copyright more restrictive than CC-BY, please include a copy, in both the original language and English, as Supporting Information. In addition please provide additional information regarding the interview guide development process, including the theories or frameworks which were employed.

Information added and copy of survey added

This study is not qualitative study but a questioner base study about demographic , dietary and risk factor

We note that you have reported significance probabilities of 0 in places. Since p=0 is not strictly possible, please correct this to a more appropriate limit, eg 'p<0.0001'.

done

3. In your Data Availability statement, you have not specified where the minimal data set underlying the results described in your manuscript can be found. PLOS defines a study's minimal data set as the underlying data used to reach the conclusions drawn in the manuscript and any additional data required to replicate the reported study findings in their entirety. All PLOS journals require that the minimal data set be made fully available. For more information about our data policy, please see <http://journals.plos.org/plosone/s/data-availability>.

Upon re-submitting your revised manuscript, please upload your study’s minimal underlying data set as either Supporting Information files or to a stable, public repository and include the relevant URLs, DOIs, or accession numbers within your revised cover letter. For a list of acceptable repositories, please see <http://journals.plos.org/plosone/s/data-availability#loc-recommended-repositories>. Any potentially identifying patient information must be fully anonymized.

Important: If there are ethical or legal restrictions to sharing your data publicly, please explain these restrictions in detail. Please see our guidelines for more information on what we consider unacceptable restrictions to publicly sharing data: <http://journals.plos.org/plosone/s/data-availability#loc-unacceptable-data-access-restrictions>. Note that it is not acceptable for the authors to be the sole named individuals responsible for ensuring data access.

We will update your Data Availability statement to reflect the information you provide in your cover letter.

Data included in the supplementary file

4. Thank you for stating the following in the Financial Disclosure section:

"The author(s) received no specific funding for this work."

We note that one or more of the authors are employed by a commercial company: Ambulatory Healthcare Services, Abu Dhabi Health services company.

All governmental organization. no commercial company, and no fundaing

a. Please provide an amended Funding Statement declaring this commercial affiliation, as well as a statement regarding the Role of Funders in your study. If the funding organization did not play a role in the study design, data collection and analysis, decision to publish, or preparation of the manuscript and only provided financial support in the form of authors' salaries and/or research materials, please review your statements relating to the author contributions, and ensure you have specifically and accurately indicated the role(s) that these authors had in your study. You can update author roles in the Author Contributions section of the online submission form.

Please also include the following statement within your amended Funding Statement. 
“The funder provided support in the form of salaries for authors [insert relevant initials], but did not have any additional role in the study design, data collection and analysis, decision to publish, or preparation of the manuscript. The specific roles of these authors are articulated in the ‘author contributions’ section.”
If your commercial affiliation did play a role in your study, please state and explain this role within your updated Funding Statement.

No funding provided for conducting the study , All the author working in the government organization .

b. Please also provide an updated Competing Interests Statement declaring this commercial affiliation along with any other relevant declarations relating to employment, consultancy, patents, products in development, or marketed products, etc.

Within your Competing Interests Statement, please confirm that this commercial affiliation does not alter your adherence to all PLOS ONE policies on sharing data and materials by including the following statement: "This does not alter our adherence to  PLOS ONE policies on sharing data and materials.” (as detailed online in our guide for authors <http://journals.plos.org/plosone/s/competing-interests>) . If this adherence statement is not accurate and  there are restrictions on sharing of data and/or materials, please state these. Please note that we cannot proceed with consideration of your article until this information has been declared.

Please include both an updated Funding Statement and Competing Interests Statement in your cover letter. We will change the online submission form on your behalf.

Not applicable. No specific funding for the study was received. All decisions regarding design, analysis, and reporting were solely made by the authors. None of the authors has any commercial affiliation

No commercial affiliation and no funding , all the author working in governmental organization

5. Your ethics statement should only appear in the Methods section of your manuscript. If your ethics statement is written in any section besides the Methods, please delete it from any other section.

Done


Reviewers' comments:

Reviewer's Responses to Questions

**Comments to the Author**

1. Is the manuscript technically sound, and do the data support the conclusions?

The manuscript must describe a technically sound piece of scientific research with data that supports the conclusions. Experiments must have been conducted rigorously, with appropriate controls, replication, and sample sizes. The conclusions must be drawn appropriately based on the data presented.

Reviewer #1: Yes

Reviewer #2: Yes

2. Has the statistical analysis been performed appropriately and rigorously?

Reviewer #1: I Don't Know

Reviewer #2: Yes

3. Have the authors made all data underlying the findings in their manuscript fully available?

The [PLOS Data policy](http://www.plosone.org/static/policies.action#sharing) requires authors to make all data underlying the findings described in their manuscript fully available without restriction, with rare exception (please refer to the Data Availability Statement in the manuscript PDF file). The data should be provided as part of the manuscript or its supporting information, or deposited to a public repository. For example, in addition to summary statistics, the data points behind means, medians and variance measures should be available. If there are restrictions on publicly sharing data—e.g. participant privacy or use of data from a third party—those must be specified.

Reviewer #1: Yes

Reviewer #2: Yes

4. Is the manuscript presented in an intelligible fashion and written in standard English?

PLOS ONE does not copyedit accepted manuscripts, so the language in submitted articles must be clear, correct, and unambiguous. Any typographical or grammatical errors should be corrected at revision, so please note any specific errors here.

Reviewer #1: Yes

Reviewer #2: Yes

5. Review Comments to the Author

Please use the space provided to explain your answers to the questions above. You may also include additional comments for the author, including concerns about dual publication, research ethics, or publication ethics. (Please upload your review as an attachment if it exceeds 20,000 characters)

Reviewer #1: The title must be changed because it is not clear

Done
Material and Methods:
I suggest to begin by stating the study design and where the study was performed. Please, specify all inclusion and exclusion criteria.
Please, specify were the study was conducted.

Done

**Study design:** Cross-sectional retrospective observational study conducted in Ambulatory health care services, Abu Dhabi, United Arab Emirates .

**Subject:** Adults aged ≥18 years presenting to Ambulatory Health Care clinic for the Weqaya screening program between October 2011 and February 2013 were randomly recruited. The Inclusion Criteria are participants who did the Weqaya screening test and agreed to be enrolled in the study after taking verbal consent through the phone. The only exclusion criteria were refusing to participate, impossibility to reach by phone, and having chronic diarrhea, chronic liver disease, and chronic kidney disease, i.e. conditions that may severely affect Vitamin D levels

Please, specify the instrumentation for immunoturbidimetry assay.
Please, verify that the following sentence is correct “Cardiac C-Reactive Protein (Latex) High Sensitive kit (CRPHS)”.
In the material and methods, Authors should state that there was also a control cohort (non-HTN) specifying the characteristic of such population, including the criteria of exclusion and inclusion

Not in our paper ?? kindly clarify

The discussion should be better organized and also more appropriate terms should be used.

Done modification in Discussion done

Reviewer #2: The manuscript cover a large part of Vitamin D deficiency issus, addressing many questions in an elegant fashion and a clear presentation. However, some minor changes could improve it: please, revise the reference list according to the major works thatare available in the literature, mainly consider citing: -PMID: 18516267 -PMID: 31330127 -PMID: 28801380 -PMID: 30588177 -PMID: 31284484 -PMID: 30793054 -PMID: 31142227

Thank you for the reference , I citaed the article related to our objective and Discussion modified

6. PLOS authors have the option to publish the peer review history of their article ([what does this mean?](https://journals.plos.org/plosone/s/editorial-and-peer-review-process#loc-peer-review-history)). If published, this will include your full peer review and any attached files.


If you choose “no”, your identity will remain anonymous but your review may still be made public.


**Do you want your identity to be public for this peer review?** For information about this choice, including consent withdrawal, please see our [Privacy Policy](https://www.plos.org/privacy-policy).

Reviewer #1: No

Reviewer #2: No
